# Detection and Characterization of ESBL-Producing *Escherichia coli* From Humans and Poultry in Ghana

**DOI:** 10.3389/fmicb.2018.03358

**Published:** 2019-01-15

**Authors:** Linda Falgenhauer, Can Imirzalioglu, Kwabena Oppong, Charity Wiafe Akenten, Benedikt Hogan, Ralf Krumkamp, Sven Poppert, Vinzent Levermann, Oliver Schwengers, Nimako Sarpong, Ellis Owusu-Dabo, Jürgen May, Daniel Eibach

**Affiliations:** ^1^Institute of Medical Microbiology, Justus Liebig University Giessen, Giessen, Germany; ^2^German Center for Infection Research, Partner Site Giessen-Marburg-Langen, Giessen, Germany; ^3^Kumasi Centre for Collaborative Research in Tropical Medicine, Kumasi, Ghana; ^4^Infectious Disease Epidemiology, Bernhard Nocht Institute for Tropical Medicine, Hamburg, Germany; ^5^German Center for Infection Research, Partner Site Hamburg-Lübeck-Borstel-Riems, Hamburg, Germany; ^6^Swiss Tropical and Public Health Institute, Basel, Switzerland; ^7^University of Basel, Basel, Switzerland

**Keywords:** *Escherichia coli*, Ghana, microbial drug resistance, poultry, extended spectrum β-lactamases (ESBL), transmission

## Abstract

**Introduction:** The increasing incidence of infections caused by extended-spectrum beta-lactamase (ESBL)-producing *Escherichia coli* in sub-Saharan Africa is of serious concern. Studies from countries with a highly industrialized poultry industry suggest the poultry production-food-consumer chain as a potential transmission route. In Africa, integrated studies at this human–animal interface are still missing.

**Aim:** To determine the molecular epidemiology of ESBL-producing *E. coli* from the intestinal tract of humans and poultry in rural Ghana.

**Methods:** During a 6-month period, fecal samples from all children admitted to the Agogo Hospital (Ghana) and broilers at eight poultry farms located within the hospital catchment area were collected. After screening on selective ESBL agar, whole genome sequencing (WGS) was performed on all ESBL isolates. The genomes were analyzed using multilocus sequence typing (MLST), ESBL genotyping and genome-based phylogenetic analyses.

**Results:** Of 140 broilers and 54 children, 41 (29%) and 33 (61%) harbored ESBL *E. coli*, respectively, with prevalences on farms ranging between 0 and 85%. No predominant sequence type (ST) was detected among humans. ST10 was most prevalent among broilers (*n* = 31, 69%). The ESBL gene *bla*_CTX-M-15_ was predominant among broilers (*n* = 43, 96%) and humans (*n* = 32, 97%). Whole-genome-based phylogenetic analysis revealed three very closely related broiler/human isolate clusters (10% of ESBL isolates) with chromosomal and plasmid-mediated ESBL genes.

**Conclusion:** The findings demonstrate a high frequency of intestinal ESBL-producing *E. coli* in rural Ghana. Considering that animal and human samples are independent specimens from the same geographic location, the number of closely related ESBL isolates circulating across these two reservoirs is substantial. Hence, poultry farms or meat products might be an important source for ESBL-producing bacteria in rural Ghana leading to difficult-to-treat infections in humans.

## Introduction

The inappropriate use of antibiotics, not only in human medicine but also in animal husbandry, has been considered a main driver leading to the increase of multidrug-resistant bacteria (Collignon et al., 2013; Chantziaras et al., 2014). Consequently, in Europe, the European Food Safety Authority (EFSA) and the European Medicines Agency (EMA) recently advocated measures to reduce use of antimicrobial agents in animal husbandry in the European Union (Murphy et al., 2017). Food-producing animals, especially poultry, have been suggested as a potential source for transmission of extended-spectrum beta-lactamase (ESBL)-producing bacteria to humans, either by direct contact or consumption of contaminated meat products, leading to the colonization of the intestinal tract and eventually to severe infections (Lazarus et al., 2014).

A number of previous studies on the inter-sectoral transmission of ESBL-producing bacteria did only use methods with low discriminatory power, which target a small number of genes (e.g., ESBL genotyping, multilocus sequence typing), and therefore may suggest, but not prove clonal transmission events (Leverstein-van Hall et al., 2011; Overdevest et al., 2011; Kluytmans et al., 2013; Lazarus et al., 2014; Valentin et al., 2014; Dahms et al., 2015). As an example, a Dutch study could not confirm previously suggested clonal transmission of ESBL-producing *E. coli* isolates between humans and poultry, when using the same collection of isolates but changing the typing method to a whole genome sequencing (WGS) approach. Instead, results rather demonstrated that antibiotic resistance between different reservoirs is likely to be transmitted by the spread of plasmids (de Been et al., 2014). Similarly, a Swedish study estimates that less than 0.1% of the Swedish population carry poultry-associated ESBL-producing isolates, but 5% are colonized with ESBL-encoding plasmids identical to those found in isolates from chicken meat and poultry (Börjesson et al., 2016).

Those findings may not be directly transferable to developing countries. In these regions, the inappropriate use of veterinary antibiotics, which are often readily sold in shops and markets without prescriptions, is considered to be very high, potentially selecting for antibiotic resistance (Mainda et al., 2015). Nevertheless, a recent review found a lower prevalence of ESBL/pAmpC *E. coli* among poultry meat products in African countries (average 16.3%) compared to reports from many European countries such as Spain (84–93%) and the Netherlands (77%) (Alonso et al., 2017). In comparison to industrialized countries, it can be assumed that inter-host transmission is more likely to happen in rural areas of sub-Saharan Africa with mainly subsistence-based agricultural communities, where people frequently live in close contact with livestock animals (Alonso et al., 2017). However, bacterial transmission among poultry and humans has not been adequately addressed in this region until now (Alonso et al., 2017).

This study aims to compare ESBL-producing *E. coli* found in the intestinal tract of humans and poultry using highly discriminatory WGS methods in order to assess potential transmission routes in a rural community of Central Ghana.

## Materials and Methods

### Study Site and Sample Collection

The study was conducted in Agogo, a town with 32,000 inhabitants situated in the Asante Akim North District within the Ashanti Region of Ghana. Between January and June 2015, all children below 15 years of age living in Agogo and nearby surroundings, who were admitted to the Agogo Presbyterian Hospital, were recruited into the study. If available, a stool sample was collected at the day of admission and transported to the hospital laboratory within 2–4 h.

A total of eight poultry farms, with around 250–1,000 chickens on each farm, are situated within and around the town of Agogo. The infrastructure with wooden shelters and open sides is comparable on all farms. Between February and April 2015, all eight farms were visited once and six farms were visited a second time between May and June 2015. On each farm visit, 10 single fecal droppings were collected with an eSwab^TM^ (Hain Lifescience, Nehren, Germany) and transported to the laboratory within 2–4 h.

The Committee on Human Research, Publications and Ethics, School of Medical Science, Kwame Nkrumah University of Science and Technology, Kumasi, Ghana and the Ethics Committee of the Ärztekammer Hamburg, Germany approved the study design and the informed consent procedures. All participants were informed about the study’s purpose and procedures in accordance with the Declaration of Helsinki. Written informed consent was obtained from the parents or the guardian on behalf of the study children prior to enrolment.

### ESBL Detection and Antibiotic Susceptibility Testing

On arrival in the laboratory, each fecal sample was directly inoculated on two selective MacConkey agar plates containing 1 mg/L ceftazidime and 1 mg/L cefotaxime, respectively. Plates were incubated at 37°C for 24–48 h in normal atmosphere. All colonies with typical *E. coli* morphology were selected and confirmed biochemically as *E. coli* by API 20E tests (bioMérieux, Marcy L’Etoile, France). For all *E. coli*, ESBL-production was confirmed by the combined disk test with cefotaxime and ceftazidime alone and in combination with clavulanic acid (Becton, Dickinson and Company, Sparks, MD, United States) as described before by the EUCAST [EUCAST guideline on detection of resistance mechanisms v 1.0 (2013-12-11)]. Quality control for each batch of cephalosporin-containing MacConkey agar plates was performed with *E. coli* ATCC 25922 and a *bla*_CTX-M-15_ positive *E. coli* isolate.

### Whole Genome Sequencing

*E. coli* isolates were stored at -80°C in cryobank tubes (Pro-lab Diagnostics, Richmond Hill, ON, Canada) and shipped on dry ice to Germany, where WGS was performed for all ESBL-producing *E. coli* isolates. In brief, whole genome DNA was isolated using the Purelink Genome DNA Mini kit (Invitrogen, Darmstadt, Germany) according to the manufacturer’s instruction. Sequencing was carried out using an Illumina Nextera XT library with 2 × 300 bp paired-end reads on an Illumina MiSeq instrument (Illumina, San Diego, CA, United States) achieving an average of 2,170,898 reads per isolate (61× average coverage). The raw data was assembled using SPAdes (version 3.0). Multilocus sequence type (ST) determination was performed using MLST 1.0 (Zankari et al., 2013). Antibiotic resistance genes were identified using ResFinder (Zankari et al., 2012) and the genetic location of the ESBL-genes was investigated using the ESBL-gene containing contigs by blastn (Altschul et al., 1990) using the nt database. Contigs depicting hits only to chromosomal sequences were considered as chromosomal hits, while those depicting hits to plasmids were considered as plasmid hits. Contigs depicting hits to both the chromosome and plasmids were considered as “ambiguous.”

The single nucleotide polymorphisms (SNPs) were called by SAMtools (Li, 2011). The tree was generated using FastTree 2 (Price et al., 2010) and drawn by Evolview (He et al., 2016).

To analyze possible transmission, isolates depicting an identical ST and present in both reservoirs were analyzed in more detail. SNPs were called as mentioned above, and regions of high recombination were excluded manually.

The raw sequencing data are available at the European Nucleotide Archive (ENA) under the project accession number PRJEB26592.

## Results

### Prevalence of ESBL-Producing Isolates in Hospitalized Children and Broilers

Between January and June 2015, 54 hospitalized children, with a median age of 20 months (IQR: 11–36 months), provided a stool sample at admission, regardless of the admission diagnosis. All children had contact to free-roaming chickens in town, but had no contact or access to the poultry farms. Chicken meat is part of the regular diet. Of all children, 33 (61%) harbored an ESBL-producing *E. coli*. From one child, two different *E. coli* were isolated, leading to a total of 34 ESBL-producing *E. coli*.

**FIGURE 1:**
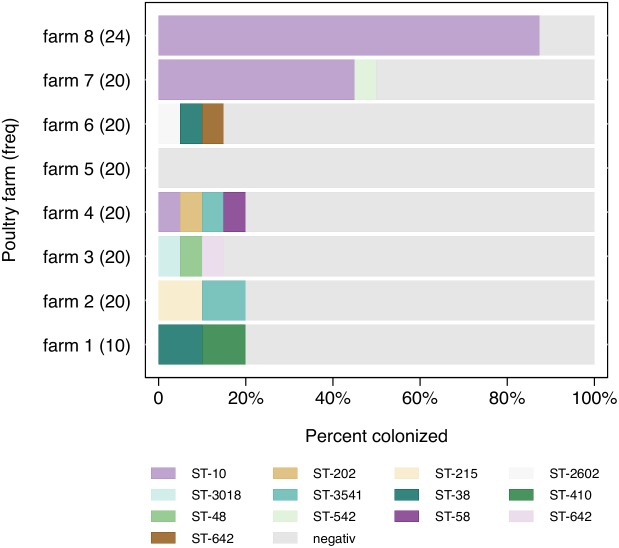
Distribution of ESBL-producing *Escherichia coli* sequence types (ST) (*n* = 45) among farms.

During the same time-period, fecal samples were obtained from 140 broilers, of which 41 (29%) were positive for an ESBL-producing *E. coli*. Prevalences differed widely between farms, ranging from 0 to 85% (Figure 1). Four broilers were detected with two different *E. coli* isolates, which results in 45 ESBL-producing *E. coli* isolates from broilers.

### Multilocus Sequence Determination of the ESBL-Producing Isolates

The 34 human isolates displayed 22 different sequence types (ST). ST-131 (12%; *n* = 4), ST-167 (9%; *n* = 3) and ST-617 (9%; *n* = 3) were most frequently detected. No clusters with more than two isolates with the same ST during a 1-month period were identified (Table 1).

**Table 1:** Frequency of sequence type (ST) in human and poultry samples (sorted by ST frequency).

STs	Human samples (*n*)	Poultry samples (*n*)	Detected in human and poultry samples
ST-10	0	31	
ST-131	4	0	
ST-38	2	2	x
ST-6359	2	1	x
ST-3541	1	2	x
ST-167	3	0	
ST-617	3	0	
ST-3268	2	0	
ST-3018	1	1	x
ST-46	2	0	
ST-58	1	1	x
ST-443	2	0	
ST-2602	0	1	
ST-295	1	0	
ST-542	0	1	
ST-940	1	0	
ST-642	0	1	
ST-34	1	0	
ST-44	1	0	
ST-90	1	0	
ST-202	0	1	
ST-4450	1	0	
ST-2141	1	0	
ST-48	0	1	
ST-155	1	0	
ST-1706	1	0	
ST-215	0	1	
ST-410	0	1	
ST-773	1	0	
ST-648	1	0	


*STs detected in both humans and poultry samples are indicted.*

Among all broiler isolates (*n* = 45), 13 different STs were identified, with ST-10 being most prevalent (69%; *n* = 31), although ST-10 has been only detected on three farms. On farm 7 and 8, ST-10 comprised 90% (*n* = 9) and 100% (*n* = 21) of all ESBL-producing isolates, respectively (Figure 1). There was no reported exchange of broilers, feed or personnel between those two farms. All other STs were not detected more than twice on each farm.

The overlap of sequence types between the two different sources (human and broiler) involved five sequence types (ST-38, ST-58, ST-3018, ST-3541, ST-6359), comprising seven broiler and seven human isolates (Table 1).

### Presence and Location of ESBL Genes

The most common ESBL genes in *E. coli* from both populations were *bla*_CTX-M-15_ (*n* = 76) followed by *bla*_SHV -12_ (*n* = 2) and *bla*_CTX-M-14_ (*n* = 1). The ESBL gene *bla*_CTX-M-15_ was equally distributed in both populations, whereas *bla*_SHV -12_ was exclusively found in broiler and *bla*_CTX-M-14_ in human isolates, respectively (Table 2). The location of the ESBL genes differed between human and broiler isolates. Among human isolates, 29% (*n* = 10) harbored plasmid and 44% (*n* = 15) chromosomally inserted ESBL genes, while the exact location could not be identified among the remaining 26% (*n* = 9). Among broiler isolates, 82% (*n* = 36) harbored a chromosomally inserted ESBL gene (Table 2). Of note, all but one (C105F) of the 31 ST-10 isolates, which were isolated from three different farms, harbored *bla*_CTX-M-15_ at an identical chromosomal location (Supplementary Table 1), indicating the spread of a distinct clone on these farms.

**Table 2:** Characteristics of the sequenced *Escherichia coli* isolates.

Isolate	Source	MLST	ESBL gene	Location of ESBL gene
C011F	Chicken	10	*bla*_CTX-M-15_	Chromosome
C012F	Chicken	10	*bla*_CTX-M-15_	Chromosome
C013F	Chicken	10	*bla*_CTX-M-15_	Chromosome
C014F	Chicken	10	*bla*_CTX-M-15_	Chromosome
C015F	Chicken	10	*bla*_CTX-M-15_	Chromosome
C016_1F	Chicken	10	*bla*_CTX-M-15_	Chromosome
C016_2F	Chicken	10	*bla*_CTX-M-15_	Chromosome
C017F	Chicken	10	*bla*_CTX-M-15_	Chromosome
C018F	Chicken	10	*bla*_CTX-M-15_	Chromosome
C019F	Chicken	10	*bla*_CTX-M-15_	Chromosome
C028F	Chicken	3541	*bla*_CTX-M-15_	Chromosome
C029F	Chicken	58	*bla*_SHV -12_	Plasmid
C050F	Chicken	3018	*bla*_CTX-M-15_	Plasmid
C060F	Chicken	38	*bla*_CTX-M-15_	Plasmid
C065F	Chicken	542	*bla*_CTX-M-15_	Ambiguous
C068F	Chicken	10	*bla*_CTX-M-15_	Chromosome
C071F	Chicken	3541	*bla*_CTX-M-15_	Chromosome
C075F	Chicken	215	*bla*_CTX-M-15_	Chromosome
C081F	Chicken	38	*bla*_CTX-M-15_	Chromosome
C088F	Chicken	410	*bla*_CTX-M-15_	Plasmid
C091_1F	Chicken	10	*bla*_CTX-M-15_	Chromosome
C091_2F	Chicken	10	*bla*_CTX-M-15_	Chromosome
C092F	Chicken	10	*bla*_CTX-M-15_	Chromosome
C093_1F	Chicken	10	*bla*_CTX-M-15_	Chromosome
C093_2F	Chicken	10	*bla*_CTX-M-15_	Chromosome
C095F	Chicken	10	*bla*_CTX-M-15_	Chromosome
C096F	Chicken	10	*bla*_CTX-M-15_	Chromosome
C097F	Chicken	10	*bla*_CTX-M-15_	Chromosome
C099_1F	Chicken	10	*bla*_CTX-M-15_	Chromosome
C099_2F	Chicken	10	*bla*_CTX-M-15_	Chromosome
C100F	Chicken	10	*bla*_CTX-M-15_	Chromosome
C105F	Chicken	10	*bla*_CTX-M-15_	Chromosome
C106F	Chicken	202	*bla*_CTX-M-15_	Plasmid
C111F	Chicken	10	*bla*_CTX-M-15_	Chromosome
C113F	Chicken	10	*bla*_CTX-M-15_	Chromosome
C114F	Chicken	10	*bla*_CTX-M-15_	Chromosome
C115F	Chicken	10	*bla*_CTX-M-15_	Chromosome
C116F	Chicken	10	*bla*_CTX-M-15_	Chromosome
C118F	Chicken	10	*bla*_CTX-M-15_	Chromosome
C119F	Chicken	10	*bla*_CTX-M-15_	Chromosome
C120F	Chicken	10	*bla*_CTX-M-15_	Chromosome
C124F	Chicken	2602	*bla*_CTX-M-15_	Plasmid
C125F	Chicken	642	*bla*_CTX-M-15_	Plasmid
C132F	Chicken	6359	*bla*_CTX-M-15_	Chromosome
C137F	Chicken	48	*bla*_SHV -12_	Plasmid
701460	Human	44	*bla*_CTX-M-15_	Plasmid
701499	Human	131	*bla*_CTX-M-14_	Chromosome
701518	Human	167	*bla*_CTX-M-15_	Plasmid
701519	Human	3541	*bla*_CTX-M-15_	Chromosome
701533	Human	131	*bla*_CTX-M-15_	Chromosome
701538	Human	617	*bla*_CTX-M-15_	Plasmid
701571	Human	131	*bla*_CTX-M-15_	Chromosome
701592	Human	46	*bla*_CTX-M-15_	Plasmid
701611	Human	38	*bla*_CTX-M-15_	Chromosome
701625	Human	617	*bla*_CTX-M-15_	Plasmid
701670	Human	295	*bla*_CTX-M-15_	Plasmid
701674	Human	2141	*bla*_CTX-M-15_	Ambiguous
701684	Human	46	*bla*_CTX-M-15_	Plasmid
701698	Human	3018	*bla*_CTX-M-15_	Plasmid
701744	Human	443	*bla*_CTX-M-15_	Ambiguous
701769	Human	773	*bla*_CTX-M-15_	Ambiguous
701790	Human	3268	*bla*_CTX-M-15_	Plasmid
701805	Human	58	*bla*_CTX-M-15_	Ambiguous
701846	Human	617	*bla*_CTX-M-15_	Ambiguous
701853	Human	167	*bla*_CTX-M-15_	Plasmid
701856	Human	940	*bla*_CTX-M-15_	Plasmid
701861	Human	6359	*bla*_CTX-M-15_	Chromosome
701867	Human	6359	*bla*_CTX-M-15_	Chromosome
701872	Human	1706	*bla*_CTX-M-15_	Plasmid
701874	Human	3268	*bla*_CTX-M-15_	Plasmid
701875	Human	155	*bla*_CTX-M-15_	Ambiguous
701889	Human	443	*bla*_CTX-M-15_	Ambiguous
701902	Human	90	*bla*_CTX-M-15_	Plasmid
701903	Human	131	*bla*_CTX-M-15_	Ambiguous
701495_1	Human	38	*bla*_CTX-M-15_	Chromosome
701495_2	Human	648	*bla*_CTX-M-15_	Chromosome
701572_1	Human	34	*bla*_CTX-M-15_	Ambiguous
701842_1	Human	4450	*bla*_CTX-M-15_	Chromosome
701887_1	Human	167	*bla*_CTX-M-15_	Plasmid


### Single Nucleotide Polymorphism (SNP) Detection in Isolates With Identical ST in Humans and Broilers

**FIGURE 2:**
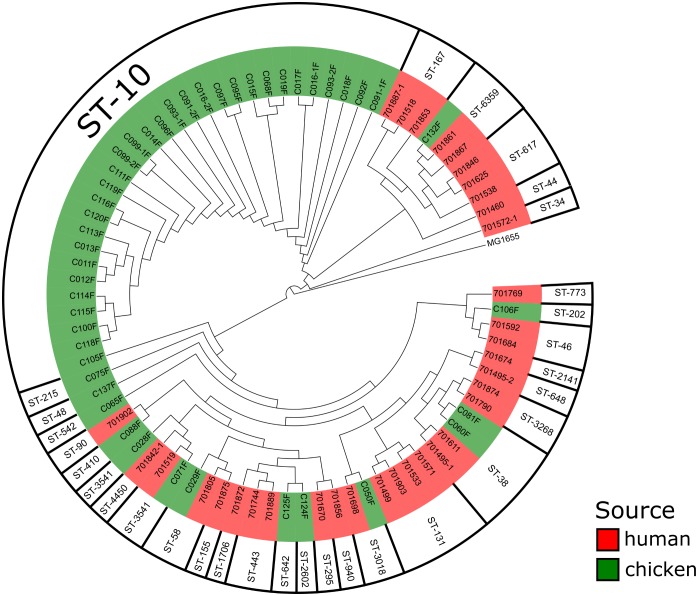
Whole genome-based phylogenetic analysis of the ESBL-producing *Escherichia coli* using *Escherichia coli* MG1655 as a reference strain.

Five human/broiler isolate clusters depicting an identical ST were regarded as possible clonal spread events and therefore investigated for SNPs (Figure 2). The ST-3541 isolates (C28F/C071F/701519) revealed a difference in 49 and 24 SNPs, respectively. In all three isolates, the *bla*_CTX-M-15_ gene was located on the chromosome at an identical position, thus indicating a common origin of these isolates.

An almost identical situation was found in the ST-6359 isolates (C132F/701861/701867). The human isolate 701861 differed by 99 SNPs from C132F, whereas 701867 differed by 17 SNPs, implying close genetic relation to the 701867 isolate. Interestingly, all ST-6359 isolates harbored *bla*_CTX-M-15_ at an identical chromosomal location, implying a common ancestor of these isolates. Similarly, the ST-3018 pair (C50F/701698) differed by only 21 SNPs and harbored *bla*_CTX-M-15_ on a plasmid.

A complete different pattern was detected in the ST-58 (C29F/701805) and ST-38 isolates (C060/C081F/701495-1/701611). ST-58 isolates differed by 4449 SNPs, thereby indicating that these are two genetically not related isolates and no transmission occurred. ST-38 isolates differed already in the chicken isolates (2034 SNPs), while the human isolates depicted 9588 (701495-1) and 9560 (701611) SNPs, respectively. This indicates that ST-38 isolates are highly diverse.

## Discussion

The study demonstrates genetic links of ESBL-producing *E. coli* that may indicate transmission between the poultry and human population in rural Ghana. With four human and four broiler isolates being closely related, 10% of all ESBL-producing isolates in this study may have been transmitted at a certain point in time between the two different populations.

The potential risk for the transmission of antimicrobial-resistant bacteria between animals or animal food products and humans in sub-Saharan Africa has been highlighted before, however, from this region no data on the clonal spread between human and animal reservoirs has been published (Soge et al., 2006; Alonso et al., 2017). Other studies using MLST-based typing methods or ESBL-genotyping do not allow to conclusively interpret the clonal spread of ESBL-producing *E. coli* (Leverstein-van Hall et al., 2011; Overdevest et al., 2011; Kluytmans et al., 2013). More recently, a Dutch study using WGS methods concluded that clonal transfer between humans and poultry of antibiotic resistant *E. coli* occurs less frequently than the transfer of resistance plasmids between the two reservoirs (de Been et al., 2014). Similarly, studies from Romania, Sweden and the United Kingdom found evidence for the transmission of ESBL-harboring plasmids instead of clonal transmission of isolates between poultry and humans (Stokes et al., 2012; Börjesson et al., 2013; Maciuca et al., 2015).

In contrast, the present results suggest clonal transmission between the two reservoirs in a rural Ghanaian town, where children were sampled at hospital admission, independent from any contact to the poultry farms to which children have no access. Taking into account the low level of sanitary and hygiene conditions in rural Ghana, inter-host transmission of bacteria is expected to be more predominant compared to industrialized countries and not necessarily limited to farm workers. Furthermore, transmission via consumption of meat products has been suggested as a potential source of multidrug resistant bacteria in Africa (Alonso et al., 2017; Eibach et al., 2018).

The high rate of ESBL-producing *E. coli* on farms is in sharp contrast to previous Ghanaian data from 2009, where no ESBL-producing *E. coli* isolates (*n* = 103) were found in broilers from Accra (Donkor et al., 2012). However, this previous study did not use any ESBL screening plates for the detection of *E. coli*, which might underestimate the ESBL production.

Notably, a high diversity of STs has been observed, with only 5 out of 30 STs overlapping between the two sampling groups. ST-10 clearly predominates within two farms. Indeed, ST-10 ESBL-producing *E. coli* have been frequently identified worldwide in the chicken production chain, including reports from Nigeria and Tanzania (Ojo et al., 2016; Seni et al., 2016). Interestingly, studies from the Netherlands, Sweden and Vietnam detected ESBL-producing ST-10 *E. coli* not only in chickens, but also in high numbers among humans (Huijbers et al., 2014; Ueda et al., 2015; van Hoek et al., 2015; Börjesson et al., 2016). However, in the present study ST-10 could not be detected among children.

In this study, ST-131, although not very frequent (*n* = 4), is the most prevalent *E. coli* subtype among children (12%). ST-131 *E. coli* have been described as an clinically relevant multidrug-resistant ST, frequently harboring *bla*_CTX-M-15_ and fluoroquinolone resistance (Mathers et al., 2015). High virulence leading to serious infections, such as invasive bloodstream, urinary tract and intra-abdominal infections have been attributed to ST131, also in Ghana (Eibach et al., 2016). Although identified in different animal reservoirs, the ST131 lineage seems to be specifically adapted to the human host and reservoirs to be rather unclear (Platell et al., 2011; Mathers et al., 2015). It has been suggested that due to acquired virulence factors ST131 is responsible for the global increase of *E. coli* harboring *bla*_CTX-M-15_ (Mathers et al., 2015).

The CTX-M-15 β-lactamase is the most prevalent ESBL type among chicken (96%) and human (97%) isolates in this study. While in many countries CTX-M-15 is one of the most frequent ESBL types in ESBL-producing bacteria, causing human infections (Valentin et al., 2014), it showed a relatively low prevalence among bacteria isolated from fecal poultry samples in previous studies using ESBL screening plates as an isolation method in the Netherlands (0%), Belgium (2%), and the United Kingdom (12%) (Smet et al., 2008; Randall et al., 2011; Huijbers et al., 2014). Studies from Japan and China also demonstrate low prevalences (0–2%) of CTX-M-15 in fecal poultry samples (Kameyama et al., 2013; Rao et al., 2014), in contrast to most surveys of African livestock, which show a predominance of *bla*_CTX-M-15_ positive ESBL-producing *E. coli* in a recent review by Alonso et al. (2017). This geographical distribution suggests that transmission between humans and poultry may occur more frequently on the African continent.

The present study has some limitations. Firstly, it does not allow any conclusions on the transmission of plasmids, but only indicates clonal bacterial transmission between poultry and humans. Therefore, the focus on clonal transfer alone will probably underestimate the transmission of resistance genes. In addition, the collection of stool samples at hospital admission may result in a higher ESBL prevalence among children compared to the general population, due to a different health care seeking behavior and potential previous antibiotic prescriptions.

## Conclusion

Apart from a high intestinal detection frequency, this study shows that highly similar ESBL-producing *E. coli* strains circulate among the human and poultry populations within a small town in rural Ghana. Hence, poultry farms or meat products might be an important source for ESBL-producing bacteria in rural Ghana leading to difficult-to-treat infections in humans. Despite widespread agreement that integration of human and animal data is desirable for antimicrobial resistance surveillance, there is very little, if any, integration of data in most low and middle-income countries. An integrated “One Health” surveillance system would be able to monitor transmission events and detect resistant bacteria in a timely manner from both sectors.

## Data Availability Statement

The raw sequencing data are available at the European Nucleotide Archive (ENA) under the project accession number PRJEB26592. All other raw data supporting the conclusions of this manuscript will be made available by the authors, without undue reservation, to any qualified researcher.

## Author Contributions

LF, CI, SP, JM, and DE designed and coordinated the study. KO, BH, VL, NS, and EO-D conducted and supervised fieldwork. KO and CA conducted laboratory work. RK and OS performed the epidemiological and bioinformatics analysis. DE and LF wrote the first draft of the paper. All authors read and approved the final manuscript.

## Conflict of Interest Statement

The authors declare that the research was conducted in the absence of any commercial or financial relationships that could be construed as a potential conflict of interest.
