# Supplementary material for: Detection and Characterization of ESBL-Producing Escherichia coli From Humans and Poultry in Ghana
Source: Front Microbiol. 2019 Jan 15;9:3358. doi: 10.3389/fmicb.2018.03358 (PMC6340976; doi:10.3389/fmicb.2018.03358)
Supplement: Supplementary file 1 [file Table_1.docx]

**Supplementary Table 1:** Integration position and site of all chromosomal insertions**.**

| **Isolate** | **Source** | **MLST** | **ESBL gene** | **Integration position*** | **Integration site** |
| --- | --- | --- | --- | --- | --- |
| C011F | Chicken | 10 | *bla*_CTX-M-15_ | 2009794 | Intergenic (between hypothetical genes *yed*K/*yed*F) |
| C012F | Chicken | 10 | *bla*_CTX-M-15_ | 2009794 | Intergenic (between hypothetical genes *yed*K/*yed*F) |
| C013F | Chicken | 10 | *bla*_CTX-M-15_ | 2009794 | Intergenic (between hypothetical genes *yed*K/*yed*F) |
| C014F | Chicken | 10 | *bla*_CTX-M-15_ | 2009794 | Intergenic (between hypothetical genes *yed*K/*yed*F) |
| C015F | Chicken | 10 | *bla*_CTX-M-15_ | 2009794 | Intergenic (between hypothetical genes *yed*K/*yed*F) |
| C016_1F | Chicken | 10 | *bla*_CTX-M-15_ | 2009794 | Intergenic (between hypothetical genes *yed*K/*yed*F) |
| C016_2F | Chicken | 10 | *bla*_CTX-M-15_ | 2009794 | Intergenic (between hypothetical genes *yed*K/*yed*F) |
| C017F | Chicken | 10 | *bla*_CTX-M-15_ | 2009794 | Intergenic (between hypothetical genes *yed*K/*yed*F) |
| C018F | Chicken | 10 | *bla*_CTX-M-15_ | 2009794 | Intergenic (between hypothetical genes *yed*K/*yed*F) |
| C019F | Chicken | 10 | *bla*_CTX-M-15_ | 2009794 | Intergenic (between hypothetical genes *yed*K/*yed*F) |
| C028F | Chicken | 3541 | *bla*_CTX-M-15_ | 853081 | phosphoethanolamine transferase *opg*E |
| C068F | Chicken | 10 | *bla*_CTX-M-15_ | 2009794 | Intergenic (between hypothetical genes *yed*K/*yed*F) |
| C071F | Chicken | 3541 | *bla*_CTX-M-15_ | 853081 | the phosphoethanolamine transferase *opg*E |
| C075F | Chicken | 215 | *bla*_CTX-M-15_ | 1394918 | Poorly characterized gene *yna*I |
| C081F | Chicken | 38 | *bla*_CTX-M-15_ | 2126072 (not MG1655,  but CP018206.1) | intergenic (between BSZ13_10895 and BSZ13_10900) |
| C091_1F | Chicken | 10 | *bla*_CTX-M-15_ | 2009794 | Intergenic (between hypothetical genes *yed*K/*yed*F) |
| C091_2F | Chicken | 10 | *bla*_CTX-M-15_ | 2009794 | Intergenic (between hypothetical genes *yed*K/*yed*F) |
| C092F | Chicken | 10 | *bla*_CTX-M-15_ | 2009794 | Intergenic (between hypothetical genes *yed*K/*yed*F) |
| C093_1F | Chicken | 10 | *bla*_CTX-M-15_ | 2009794 | Intergenic (between hypothetical genes *yed*K/*yed*F) |
| C093_2F | Chicken | 10 | *bla*_CTX-M-15_ | 2009794 | Intergenic (between hypothetical genes *yed*K/*yed*F) |
| C095F | Chicken | 10 | *bla*_CTX-M-15_ | 2009794 | Intergenic (between hypothetical genes *yed*K/*yed*F) |
| C096F | Chicken | 10 | *bla*_CTX-M-15_ | 2009794 | Intergenic (between hypothetical genes *yed*K/*yed*F) |
| C097F | Chicken | 10 | *bla*_CTX-M-15_ | 2009794 | Intergenic (between hypothetical genes *yed*K/*yed*F) |
| C099_1F | Chicken | 10 | *bla*_CTX-M-15_ | 2009794 | Intergenic (between hypothetical genes *yed*K/*yed*F) |
| C099_2F | Chicken | 10 | *bla*_CTX-M-15_ | 2009794 | Intergenic (between hypothetical genes *yed*K/*yed*F) |
| C100F | Chicken | 10 | *bla*_CTX-M-15_ | 2009794 | Intergenic (between hypothetical genes *yed*K/*yed*F) |
| C105F | Chicken | 10 | *bla*_CTX-M-15_ | 2786126 | hypothetical gene *yga*Q |
| C111F | Chicken | 10 | *bla*_CTX-M-15_ | 2009794 | Intergenic (between hypothetical genes *yed*K/*yed*F) |
| C113F | Chicken | 10 | *bla*_CTX-M-15_ | 2009794 | Intergenic (between hypothetical genes *yed*K/*yed*F) |
| C114F | Chicken | 10 | *bla*_CTX-M-15_ | 2009794 | Intergenic (between hypothetical genes *yed*K/*yed*F) |
| C115F | Chicken | 10 | *bla*_CTX-M-15_ | 2009794 | Intergenic (between hypothetical genes *yed*K/*yed*F) |
| C116F | Chicken | 10 | *bla*_CTX-M-15_ | 2009794 | Intergenic (between hypothetical genes *yed*K/*yed*F) |
| C118F | Chicken | 10 | *bla*_CTX-M-15_ | 2009794 | Intergenic (between hypothetical genes *yed*K/*yed*F) |
| C119F | Chicken | 10 | *bla*_CTX-M-15_ | 2009794 | Intergenic (between hypothetical genes *yed*K/*yed*F) |
| C120F | Chicken | 10 | *bla*_CTX-M-15_ | 2009794 | Intergenic (between hypothetical genes *yed*K/*yed*F) |
| C132F | Chicken | 6359 | *bla*_CTX-M-15_ | 390016 | intergenic (between hypothetical proteins *yki*B and *yai*T) |
| 701499 | Human | 131 | *bla*_CTX-M-14_ | 553221 | UDP-2,3-diacylglucosamine diphosphatase *lpx*H |
| 701519 | Human | 3541 | *bla*_CTX-M-15_ | 853081 | phosphoethanolamine transferase *opg*E |
| 701533 | Human | 131 | *bla*_CTX-M-15_ | 1031902 | intergenic (between *ser*T and *hya*A) |
| 701571 | Human | 131 | *bla*_CTX-M-15_ | 1031915 | intergenic (between *ser*T and *hya*A) |
| 701611 | Human | 38 | *bla*_CTX-M-15_ | 2507981 (not MG1655, but CP022229.2) | hypothetical protein (DBR04_150H70) |
| 701861 | Human | 6359 | *bla*_CTX-M-15_ | 390016 | intergenic (between hypothetical proteins *yki*B and *yai*T) |
| 701867 | Human | 6359 | *bla*_CTX-M-15_ | 390016 | intergenic (between hypothetical proteins *yki*B and *yai*T) |
| 701495_1 | Human | 38 | *bla*_CTX-M-15_ | 2507981 (not MG1655, but CP022229.2) | hypothetical protein (DBR04_150H70) |
| 701495_2 | Human | 648 | *bla*_CTX-M-15_ | 3914467 (not MG1655, but CP023258.1) | RecQ family ATP-dependent DNA helicase (CLH66_20400) |
| 701842_1 | Human | 4450 | *bla*_CTX-M-15_ | 2422893 | recombination-promoting nuclease *rpn*B |

*relative to *E. coli* MG1655, if not stated differently
